# Healthcare Transformation: Artificial Intelligence Is the Dire Imperative of the Day

**DOI:** 10.7759/cureus.62652

**Published:** 2024-06-18

**Authors:** Abhishek Choubey, Shruti Bhargava Choubey, Prafull K, Vandana S Daulatabad, Nitin John

**Affiliations:** 1 Electronic Communication, Sreenidhi Institute of Science & Technology, Hyderabad, IND; 2 Physiology, All India Institute of Medical Sciences, Bibinagar, Hyderabad, IND

**Keywords:** healthcare application, smart health, disease prediction, healthcare techonology, artificial intelligence (ai)

## Abstract

At present, healthcare systems around the world are confronted with unprecedented challenges caused by aging demographics, increasing chronic diseases, and resource challenges. In this scenario, artificial intelligence (AI) emerges as a disruptive technology that can provide solutions to these complicated problems. This review article outlines the vital role played by AI in altering the health landscape. The constant demand for effective and accessible healthcare demands the use of new solutions. AI can be described as an important imperative, enabling advancements in many areas of the delivery of healthcare. This review article explores the possibilities of use of AI to aid in the field of healthcare assistants, diagnosing, disease prediction, and personalized treatment and the discovery of drugs, telemedicine and remote monitoring of patients, robotic-assisted procedures imaging for pathology and radiology analysis, and the analysis of genomic data. By analyzing the existing research and cases, we explain how AI-driven technology can optimize processes in healthcare, improve diagnosis accuracy, improve the quality of treatment, and simplify administrative tasks. By highlighting the most successful AI applications and laying out possible future developments, the review article will provide insight for healthcare professionals, policymakers, researchers, and other stakeholders in harnessing the power of AI to transform healthcare delivery and enhance the quality of care for patients.

## Introduction and background

Artificial intelligence (AI) has developed significantly in the field of healthcare, and the latest developments suggest that it is a transforming technology. AI comprises a wide range of analytical methods employed in healthcare settings. It includes machine learning (ML) and deep learning (DL), which are capable of quickly processing massive quantities of medical information and quickly identifying patterns within it. In the 1970s and 1980s, AI systems were often employed to carry out essential tasks, for example, analyzing medical images to assist doctors in diagnosing. AI applications have grown significantly since the latter half of 2000 and in the early 1990s. They were used to aid in finding drugs and monitoring systems for patients and medical decision-support devices and devices to assist physicians in making decisions and devices to support physician decision-making and devices to support physician decision-making [1]. In the last few years, advances in machine-learning methods, such as DL, have boosted AI's ability to accurately analyze data while making precise predictions.

AI can improve efficiency, precision, and accuracy and reduce the time that is spent managing different elements in the system. Examples include diagnostics in the study of laboratory imaging lab diagnosis and financial management. AI can streamline healthcare procedures and workflows, providing better care and more efficiency. The fundamental goal of healthcare is to enhance patient well-being. Examples of AI techniques used in healthcare contexts include ML, DL, and natural language processing (NLP) [2]. Healthcare is constantly evolving to offer patients more effective care. Collectively, administrators, doctors, front-line staff, and insurance providers develop an effective healthcare system. Pharmacies, radiology/pathology labs, pharmaceutical research labs, and other organizations collaborating to offer healthcare are examples of network providers. AI has shown itself to be a cutting-edge technology that has changed our ideas about how healthcare is delivered and created a wealth of prospects that will probably determine its future.

This article reviews AI technology used in healthcare, with special emphasis on its use to detect illnesses and communicate with patients and the creation of strategies for engaging. Human rights and ethical and legal concerns about information will be considered to enhance our understanding of how AI is used in healthcare settings. It will also assist health professionals in implementing the latest advances in technology.

AI fundamentals

AI is the capacity of computers to display intelligent behavior. It could encompass abilities to solve problems and learn in addition to decision-making and perception abilities. It is important to remember that AI is not one thing but rather encompasses multiple techniques and methodologies. Data is at the core of AI. This can come in both structured (e.g., spreadsheets) and unstructured forms (text/images). Both the quality and quantity of data significantly impact AI models' performance and potential.

Core Technologies

ML: ML is the process of allowing computers to understand data, without explicit programming [3]. There are many ML algorithms, such as supervised (learning from data labeled) and unsupervised learning (finding patterns in data that is not labeled).

DL: DL is a subfield within ML that employs artificial neural networks based on human neural structures for analyzing huge amounts of data to identify complex patterns like image recognition or the natural processing of language.

Natural language processing (NLP): NLP technology allows computers to comprehend and interpret human speech patterns, including chatbots and machine translation and sentiment analysis. It also powers applications such as chatbots.

Computer vision: Computer vision, a field of AI, is the process of providing computers with the capability to "see" and interpret their surroundings. It is the process of extracting relevant information from videos and images so that machines can recognize the objects, scenes, or other activities in these sources [4].

## Review

AI applications in healthcare

**Figure 1:**
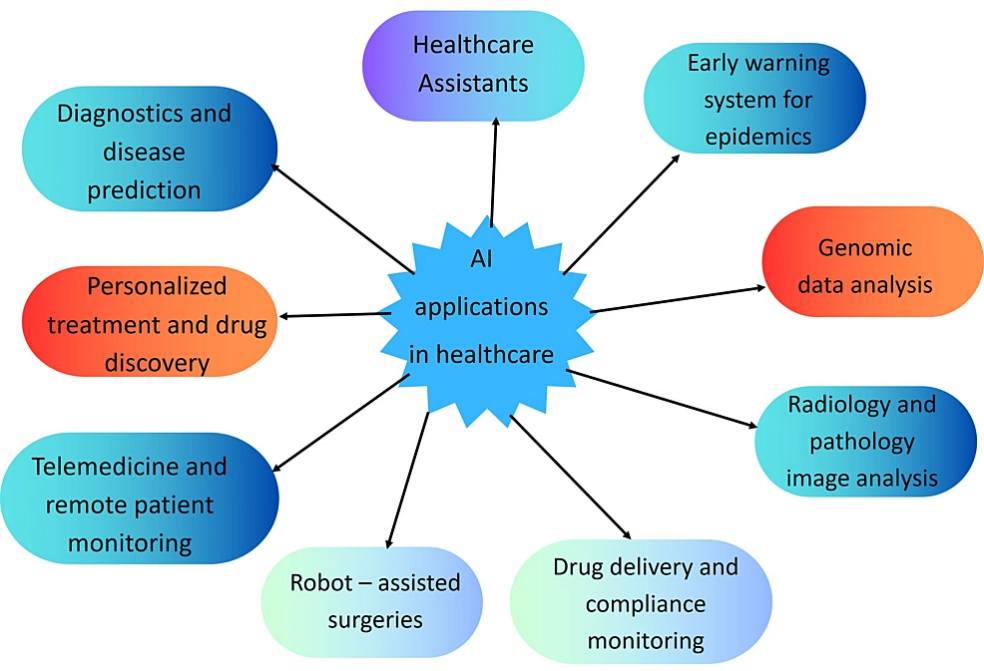
AI applications in healthcare Credits for the figure: Abhishek Choubey

AI refers to the application of technology and computers to imitate human intelligence and automate difficult jobs. The different application of AI in healthcare is shown in Figure 1. AI is revolutionizing healthcare through diagnosis, treatment, administration duties, and patient care duties. From improving efficiency to tailoring treatment plans and improving patient outcomes, AI is driving the healthcare revolution soon. AI-powered computers, which attempt to replicate the abilities humans have, can surpass humans in a range of ways, like the capability to analyze vast volumes of data within a relatively short time to identify irregular patterns, trends, and other patterns. AI can enhance a variety of medical practices, which range from identifying diseases to determining the most effective treatment options for patients with serious cancer. Surgery instruments that are equipped with AI allow surgeons to perform more efficiently when performing surgery by restricting their physical movements and providing accurate details when performing the surgery [5].

Healthcare Assistants

AI-enhanced healthcare assistants are becoming more efficient owing to AI. AI assistants could alter the care of patients and assist with many benefits for healthcare professionals and patients alike. Virtual health assistants are in charge of many tasks, including answering calls from patients and emails, keeping their medical records, while protecting confidential information setting appointments with physicians, and reminding patients of appointments for follow-up appointments and clinical meetings, among others. Patients suffering from chronic illness are being monitored remotely using wearable devices that make use of AI. AI examines the information gathered and informs healthcare professionals of any issues that could be. Wearables collect vital data like the quality of sleep and activity levels, vitals, and medicines taken. AI then analyzes these data to determine health risks and monitor progress toward reaching health goals.

Systems that make use of cognition, computing with augmented reality, and body and voice movements are combined to create this. The combination of cognitive computing with augmented reality helps stimulate and resolve complex human thinking. It is among the most beneficial AI in healthcare, providing patients with a personalized experience for managing their health and answering their concerns. Medical professionals and patients benefit from a reduced number of visits to hospitals [6].

Diagnostics and Disease Prediction

AI has become an invaluable component in healthcare diagnosis and prediction, offering unparalleled capabilities to quickly analyze complex medical records and assist healthcare providers with making accurate diagnoses more quickly and precisely. The use of AI in disease prediction and diagnostics is shown in Table 1. AI has proven its effectiveness more accurately, and based on algorithms, the prediction of cancer is easily possible as per the National Cancer Institute. The fields of AI, DL, and ML are all important for improving patient outcomes and cancer therapy. AI has emerged as a critical diagnostic and predictive tool with a significant promise to revolutionize healthcare delivery [7].

**Table 1:** AI for disease prediction and diagnostics Credits for the table: Abhishek Choubey

S. No.	Area	Descriptions
AI in disease prediction	Early detection	AI technology can support doctors in early disease identification and intervention strategies that are most likely to succeed, increasing both effectiveness and likelihood.
	Identifying risk factors	Artificial intelligence analyzes environmental, lifestyle, and genetic data to predict a person's susceptibility to certain diseases like heart disease, diabetes, and Alzheimer's.
	Personalized medicine	AI technology can assist medical professionals in establishing screening and preventative methods based on specific risk factors, resulting in improved care delivery.
AI in diagnostics	Image analysis	AI algorithms can analyze medical images like Xrays, CT scans and mammograms with high accuracy to enable rapid and precise diagnosis of ailments like cancer heart disease, and pneumonia
	Decision support	AI systems can operate as virtual assistants for doctors, delivering information and advice based on research-based medicine, enhancing efficiency, and reducing diagnostic errors.
	Data analysis	AI technology can quickly scan large amounts of patient data such as medical histories, laboratory test findings, as well as genetic profiles, to find trends and relationships that correspond to particular disorders. This approach is particularly helpful for treating complicated diseases with multiple relevant elements.

AI in disease prediction

Early Detection

AI technology can support doctors in early disease identification and intervention strategies that are most likely to succeed, increasing both effectiveness and likelihood [8].

Identifying Risk Factors

AI analyzes environmental, lifestyle, and genetic data to predict a person's susceptibility to certain diseases like heart disease, diabetes, and Alzheimer's disease [9].

Personalized Medicine

AI technology can assist medical professionals in establishing screening and preventative methods based on specific risk factors, resulting in improved care delivery [3].

AI in diagnostics

Image Analysis

AI algorithms can analyze medical images like X-rays, CT scans, and mammograms with high accuracy to enable rapid and precise diagnosis of ailments like cancer heart disease and pneumonia [10,11].

Decision Support

AI systems can operate as virtual assistants for doctors, delivering information and advice based on research-based medicine, enhancing efficiency, and reducing diagnostic errors [11].

Data Analysis

AI technology can quickly scan large amounts of patient data, such as medical histories, laboratory test findings, and genetic profiles, to find trends and relationships that correspond to particular disorders. This approach is particularly helpful for treating complicated diseases with clinical parameters.

Personalized Treatment and Drug Discovery

AI has a significant role in drug discovery and personalized medicine, creating numerous opportunities to provide more individualized care. AI can predict how a patient will react to a medication and identify any genetic differences connected to particular diseases using genetic data. They can also analyze huge quantities of Electronic health record (EHR) data, such as medical histories and lab test results, to identify patterns that indicate specific responses to treatment, which allows doctors to tailor their treatments for each patient, while AI-powered sensors and wearable devices assist in determining the most effective treatment in any situation. Wearable sensors continuously track patient health (e.g., blood sugar and heart rate), identify any potential problems or adverse side effects from treatment, and act quickly for proactive intervention and personalized adjustment to treatment strategies [12].

AI can analyze huge amounts of biological data to rapidly identify drug targets linked to certain illnesses. This allows the drug development teams to concentrate on targets that are promising and have better chances of achieving successful development, speeding up drug development. AI can rapidly scan millions of drug molecules virtually, allowing for the simulation of the way they react with biochemical targets. AI can rapidly identify promising candidates for further development, which saves both time and money by removing traditional screening techniques. AI can also evaluate current drugs to determine their effects on various ailments, opening the possibility of finding new uses of existing medicines that reduce time and money; AI also assists with creating individualized treatment plans based on the specific needs of patients and genetic profiles [13].

EHR Management

AI is changing the way we handle EHRs with unprecedented possibilities to simplify workflows, improve the quality of data, and improve the quality of care for patients; it can analyze medical record images and reports on imaging and other records to find relevant data. These data are then used to fill in EHR fields, saving doctors time and money while also reducing manual errors. AI systems can analyze patient information and provide immediate suggestions for diagnosis treatments, treatment plans, and medical safety checks to aid physicians in making educated decisions. AI-powered voice assistants can translate patient consultations with doctors straight into EHR, increasing note accuracy while freeing up the clinician's time to allow patient interaction. AI can identify crucial details from text that is not structured in EHRs and extract pertinent clinical information for study and analyses. AI can analyze large datasets across a variety of EHRs to discover patterns, anticipate outbreaks of diseases, and help inform the public about health interventions. AI models can analyze information from patients to anticipate the risk of health problems, which allows for prompt treatment and proactive measures. AI can analyze the individual patient's medical history to provide individual treatment strategies and medication regimens. AI can examine large amounts of data from EHRs to determine patterns and trends in the incidence of disease, which allows to target public health interventions [14].

Telemedicine and Remote Patient Monitoring

AI is quickly becoming an indispensable resource in the field of telemedicine and remote monitoring of patients (RPM) and is revolutionizing the delivery of healthcare services online and in time. AI-powered virtual assistants can help patients get access to healthcare faster while removing healthcare professionals from administrative burdens. AI algorithms can analyze the patient's complaints and identify possible sources, allowing the most efficient and successful treatments, while making consultations faster and more efficient. Chatbots powered with AI and virtual therapy provide basic support to mental health professionals and self-management tools to improve access to services in areas that have accessibility issues. AI can effectively analyze sensors and wearable data to spot early indicators of decline. They can then take steps to resolve issues swiftly and extend the life of patients. In addition, this advanced AI solution can provide alerts tailored specifically to an individual's health condition and treatment plans, enabling early intervention and helping prevent issues altogether. AI can efficiently analyze patient data to detect potential health hazards and take necessary measures to eliminate them. AI excels at recognizing early warning signs for diseases or complications and prompting interventions that prevent potentially life-threatening health risks [15].

 *Robot-Assisted Surgeries*

The robotic-assisted surgery may also be known as robotic surgery. Also called minimally surgical (robot-assisted implant surgery (RAIS)), it makes use of robotic technology to assist surgeons with their surgery procedures. They are fitted with surgical instruments, such as cameras, robotic arms, and cameras that can create 3D images of surgical sites. AI algorithms allow surgeons to perform complex surgical procedures with greater accuracy and control. AI-powered imaging technology gives surgeons better visualization capabilities that allow greater precision in the detection of anatomical structures and anomalies that can improve the outcome of the patient resulting in less tissue trauma and better results for the patients. AI can significantly decrease the risk of injury while also protecting tissues surrounding it from damage, leading to improved quality of life for the patient. AI-powered systems aid surgeons in the development and execution of complicated surgical procedures that require crucial indicators, such as blood flow, or other variables that require immediate feedback, such as vital signs. VR and AI-controlled simulators provide surgeons with the opportunity to develop their skills in a controlled and controlled environment and practice under less-than-ideal conditions. Furthermore, these devices aid in facilitating development and learning. This is the process of transferring knowledge, which is particularly important in complex or unusual procedures [16].

AI algorithms are built upon exact inputs of data and rigorous validation procedures to assure their safety and accuracy during surgical procedures. Algorithmic mistakes, malfunctioning hardware, and cybersecurity-related risks highlight the need for quality assurance procedures, as well as the oversight of regulators for AI techniques employed in surgery. The use of AI can also create ethical issues to consent of patients the autonomy of each patient, and their obligation to others. Specific guidelines need to be established regarding informed consent before the use of these methods in addition to concerns about potential liability in the event of negative results from its usage, and the potential for supervision by humans of the decision-making process [17].

Drug Delivery and Compliance Monitoring

AI algorithms utilize the information of patients to modify dosages and treatment schedules to meet the requirements of each patient, improving the effectiveness of treatment. AI assists healthcare professionals in recognizing patients who struggle to stick to their prescribed dosage and respond quickly to boost compliance and improve the efficacy of treatment. AI drug delivery systems track the compliance of patients in real time and offer alternatives like reminders and automated adjustments to dosages and also access to health professionals' algorithms can look at patterns in the behavior of patients to discover the causes that influence the patient's adherence to medications and aid healthcare professionals in creating specific strategies to improve compliance. Smartphone apps powered by AI help patients by reminding them that they should take their medication promptly, making sure they comply with their treatment, and providing regular updates regarding the progress of their treatment. The virtual health tools powered by AI can motivate patients to remember that they should take their medication at the right time, resolve concerns regarding treatment plans, and improve the rate of compliance. AI analytics tools aid health professionals in identifying the patterns, trends, and medication compliance between different patient groups and enabling targeted interventions to improve healthcare delivery. AI algorithms can also aid the supply chain of pharmacies to improve to ensure that medicines are readily available when they are needed and result in reduced stock levels, shortages of supplies, and better compliance for patients by facilitating accessibility [18].

Radiology and Pathology Image Analysis

Image analysis and pathology both use medical imaging technologies to detect and monitor diseases, but their methodologies differ in terms of image types studied and the level of detail provided [19]. Radiography uses imaging technologies, such as X-rays, CT scans, MRIs, and ultrasounds, to create images of organs, bones, and other tissues within the body, which may help in the detection of abnormalities, such as tumors, fractures, or bleeding; pathology examines tissue or liquid samples under a microscope for any distinctive changes associated with specific illnesses, such as changes in cell size arrangement or abnormalities within.

Table 2 presents the comparison of radiology and pathology [20].

**Table 2:** Comparison of radiology and pathology

Feature	Radiology	Pathology
Imaging technique	X-ray, CT scan, MRI, ultrasound	Microscopic examination of tissues or fluids
Level of detail	Shows organs, bones, and other tissues	Shows individual cells and tissues
Applications	Detecting tumors, fractures, infections, and other abnormalities	Diagnosing cancer, and other diseases, and monitoring treatment response

AI is fast becoming an integral component of radiography and image processing. AI algorithms can detect certain patterns within medical photographs that would be difficult for our eyes to see, which may increase the accuracy of diagnosis and efficiency.

AI systems are effective in discovering the smallest of imperfections or patterns in radiological images. They are able to spot fractures, cancers, or neurological disorders in their early stages. Early intervention and diagnosis could increase the quality of life for patients and lower the expense of medical care. Analyzing radiological images can be conducted quantitatively with the assistance of AI. This allows for an accurate examination of the anatomical structure, progress of disease, and the specific characteristics of tissues. By using analytical technology that is controlled by the data, it is an approach that reduces variation between observers and improves precision of diagnosing and accuracy. AI devices can integrate data from genetic tests and radiological scans from an electronic medical record to give a precise description of health issues patients suffer from. This enables AI systems to personalize medical care and monitoring, which can improve the capacity of the patient to lead an active and healthy life [21].

When it comes to separating different tissues and identifying the relevant details in histological images, AI algorithms are highly efficient in analyzing images to pinpoint the reason. The precise measurements for the structure and shape of cells, as well as their form and the expression of biomarkers, can be accomplished with AI algorithms that assist in the precise diagnosis and forecasting. AI-driven digital pathology instruments facilitate sharing analysis, analysis, and the remote inspection of histopathological images, which reduces geographical barriers and facilitates international collaboration among pathologists. Through these platforms, access to experts' views in areas that are not covered by the standard of service and quality control and exchange of information are improved. Decision-support tools based on AI assist pathologists in making sense of complex histopathology images. Based on the best practices for clinical practice and algorithms with a scientific foundation, they provide suggestions to diagnose, determine the risk, and suggest the best treatment options. This improves the consistency of diagnosis and accuracy, allowing pathologists to make better choices [22-23].

Early Warning System for Epidemics

An epidemic is spreading rapidly of illness in a large group of people in a particular region in a short period. It can affect many more people than is normal for the location. AI could play a crucial part in the prevention of epidemics and detection and response strategies. AI algorithms can analyze a variety of sources of data, such as social media, Internet searches, news articles, and healthcare records, to spot early signs of an outbreak. AI can detect patterns and irregularities, providing health officials with early alerts that enable them to act more swiftly. AI-powered surveillance systems use data from sources, such as digital health records and hospital admissions, as well as disease-reporting platforms, to monitor outbreaks of infectious diseases in real time. They can recognize epidemics, determine high-risk areas, and then carry out focused treatments [24-25].

Applying AI methods like ML and computational modeling, epidemiological data may be investigated to develop models to predict the spread of diseases. By including human behavior, demography, and the environment as factors in models for predicting epidemics, it is possible to predict how the disease will spread and assess how effectively prevention strategies are operating. AI-powered diagnostic technologies help doctors find diseases. With the use of ML algorithms, medical images, test results, and other clinical data can be analyzed to provide an accurate diagnosis for conditions like influenza, COVID-19, malaria, and tuberculosis. AI can help speed up the development of drugs through the rapid analysis of massive genomic, proteomic, and chemical databases to discover promising drugs. The safety and effectiveness of drugs can be assessed with the help of AI algorithms, which speed up the development of treatments and vaccines against new infectious diseases. In addition, the effective distribution of vaccination programs that ensure equal access and effectiveness is possible through AI algorithmic optimization. AI could provide suggestions to ensure that vaccination plans are effective, which reduce the risk of transmission of disease and increase the effectiveness of vaccination by incorporating transportation networks, supply chains, and the density of population. AI can analyze social media and online activity data to assess public opinion and attitudes, as well as adherence to health advice given by the public in the event of an outbreak. The information obtained is used to create effective communication strategies make healthy lifestyle choices and correct inaccurate information. Through the analysis of location information from mobile devices, AI-powered contact tracking systems will streamline the identification of individuals who are exposed to diseases. If they are first exposed, AI algorithms can then analyze their movements determine if they are screened and decide on protocols for quarantine. During this quarantine, E‑learning is paving its way into medical education and facilitators are devising newer methods to impart maximum knowledge to their students to overcome academic loss due to the COVID‑19 pandemic [26]. Those who spent an excessive amount of time on their smartphones for social contact, with an average screen time of five hours, showed signs of mild to moderate sadness, moderate anxiety, and tension, demonstrating that social media harmed the mental health of medical undergraduates [27].

Genomic Data Analysis 

The process of analyzing genomic data is the method of gathering valuable data from a living organism's vast storage of genetic material, also referred to by its genome [28]. This can provide valuable insights into the state of health or disease and their development processes. Genomic data is a term used to describe all DNA sequences found within the cell walls of an animal and within other living organisms. It encompasses the entire genes (coding regions) that direct your body to produce proteins, in addition to non-coding regions that are essential to cell-related or gene-related processes. Genomic data analysis refers to the study of effectively and efficiently the structures, development, and functions of genomes and all genetic material in an organism, to grasp and efficiently their purpose and their structure and modifications. AI has revolutionized genomic analysis using powerful tools that allow quick accurate analysis. Researchers employ AI techniques, such as hidden Markov models (HMMs) and DL models, to assess the DNA sequences or proteins and then to discover similarities and differences between them. AI technology allows researchers to find genetic variations in genomic data, such as SNPs, also known as single-nucleotide polymorphisms (SNPs) or insertions/deletions (indels), which are crucial for studying populations' genetics and disease genetics. ML algorithms developed for learning can be used to analyze genomic data to categorize it into health or disease conditions or determine traits based on genetic information. AI techniques are increasingly being used to analyze information on gene expression through techniques like RNA sequence analysis (RNA-seq). This helps researchers gain a greater understanding of how gene expression affects different biological processes. AI algorithms can also help researchers predict three-dimensional structures drawn from DNA sequences and provide insights into interactions and roles that can assist in the creation of CRISPR-based genome editing strategies by anticipating off-target consequences and increasing guide RNA sequences to achieve precise editing of genomes. AI can assist when analyzing vast genomic data sets from diverse populations to understand genetic diversity, population structure, and evolutionary relationships. AI algorithms examine medical and genomic information to find potential targets for drugs, assess responses to existing therapies, and tailor treatments based on each patient's genetic makeup. AI technologies assist in comprehending functional components in the genome, such as regulatory areas, non-coding RNAs, and epigenetic changes.

## Conclusions

AI is the most effective solution to the rising costs of healthcare and demands. AI can provide solutions to various aspects of healthcare delivery by using the most current methods, big data analytics, and computational power. AI gives healthcare professionals numerous opportunities to change the way they deliver healthcare services, from enhancing the diagnosis and treatment options to improving patient outcomes and maximizing administrative tasks. There are however particular challenges associated with the integration of AI in healthcare, including the limitations on access, privacy issues about data limitations, and the need to collaborate with other professionals.
